# Male-Specific Association between Dopamine Receptor D4 Gene Methylation and Schizophrenia

**DOI:** 10.1371/journal.pone.0089128

**Published:** 2014-02-19

**Authors:** Jia Cheng, Yunliang Wang, Kena Zhou, Lingyan Wang, Jinfeng Li, Qidong Zhuang, Xuting Xu, Leiting Xu, Kai Zhang, Dongjun Dai, Rongjiong Zheng, Guangxue Li, Aiping Zhang, Shugui Gao, Shiwei Duan

**Affiliations:** 1 Zhejiang Provincial Key Laboratory of Pathophysiology, School of Medicine, Ningbo University, Ningbo, Zhejiang, China; 2 Department of Psychiatry, Ningbo Kangning Hospital, Ningbo, Zhejiang, China; 3 Neurology Department of the 148th Hospital of PLA, Zibo, Shandong, China; 4 Bank of Blood Products, Ningbo No. 2 Hospital, Ningbo, Zhejiang, China; 5 Key Laboratory for the Genetics of Developmental and Neuropsychiatric Disorders, Bio-X Institutes, Ministry of Education, Shanghai Jiao Tong University, Shanghai, China; The George Washington University, United States of America

## Abstract

**Objective:**

The goal of our study was to investigate whether *DRD4* gene DNA methylation played an important role in the susceptibility of Han Chinese SCZ.

**Methods:**

Using the bisulphite pyrosequencing technology, DNA methylation levels of 6 CpG dinucleotides in *DRD4* CpG island were measured among 30 paranoid SCZ patients, 30 undifferentiated SCZ patients, and 30 age- and gender-matched healthy controls.

**Results:**

Strong correlation was observed among the six CpG sites (r>0.5, P<0.01), thus average methylation levels were applied thereafter. Our results indicated that there was a significant association between *DRD4* methylation and the risk of SCZ (P = 0.003), although there was no significant difference in *DRD4* methylation between the two SCZ subtypes (P = 0.670). A breakdown analysis by gender showed that the significant association of *DRD4* methylation and SCZ was driven by males (P<0.001) but not by females (P = 0.835). *DRD4* methylation was significantly associated with p300 in male SCZ patients (r = −0.543, P = 0.005) but not in female SCZ patients (r = 0.110, P = 0.599). Moreover, receiver operating characteristic (ROC) curves showed *DRD4* methylation was able to predict the status of SCZ in males [area under curve (AUC) = 0.832, P = 0.002] but not in females (AUC = 0.483, P = 0.876). Finally, a further expression experiment showed that *DRD4* methylation in the gene body was positively associated with gene expression, although the exact mechanism of gene regulation remained unknown for this interesting *DRD4* methylation.

**Conclusion:**

The gender disparity in the *DRD4* DNA methylation provides novel insights into the pathogenesis of SCZ.

## Introduction

Schizophrenia (SCZ) is a complex mental disorder with a global lifetime prevalence of about 1% [1]. Paranoid SCZ and undifferentiated SCZ are two most common subtypes of SCZ according to DSM-IV criteria [2]. The hallmark symptoms of these subtypes comprise delusions, hallucinations, extremely disorganized behavior and negative symptoms [3]. SCZ is a complex disorder resulting from both genetic and environmental factors, including genetic vulnerability, neurotoxicity, unbalanced neurotransmitter, living environment, drug abuse and prenatal stressors [4], [5], [6]. Twin and family studies have revealed that SCZ is a heritable disorder [7], [8], although heritability estimation varies due to the difficulty in separating the effects of genetics and environmental factors [9].

Dopamine (DA) is one of the most important neurotransmitters in human brain, and dysfunction of DA system is a fundamental event in SCZ development [10], [11]. An inverted-U curve can describe the relationship between DA activity and cortical function, either up or down stimulation of DA can result in poor cerebral performance [12], [10]. The dopamine receptor D4 (DRD4) is a subtype of dopamine receptor family that is activated by the DA [13]. The neuregulin and DA modulation of hippocampal function was dependent on DRD4 activation and genetically associated with SCZ [14]. It can regulate many neurological processes connecting with psychiatric disorders [15]. The variations of DRD4 were widely reported to be related to diverse human behavior phenotypes [16], [17].

Epigenetic modification is one of the mechanisms underlying the interaction between environmental exposure and individual genetic background in the development of psychiatric disorders [18], [19]. DNA methylation is a crucial way of epigenetic mechanisms that regulate expression of numerous functional genes in human nervous system [4], [18], [20]. However, there is a lack of epigenetic evidence for the involvement of *DRD4* in SCZ pathogenesis.

The p300 wave, a component of an event-related potential, is often used as metrics of cognitive function and has been proved relative to SCZ cognitive impairment [10]. The p300 waveforms of monozygotic twins are almost the same and more unanimous than that in dizygotic twins [10], [21]. This implied that p300 amplitude might be under genetic control and may serve as an endophenotype for SCZ [10]. In the present study, we explore the association between *DRD4* methylation and SCZ clinical features such as cognitive symptomatology and medication. The goal of our study is to assess the association between *DRD4* methylation and SCZ.

## Materials and Methods

### Samples and clinical data

The samples in the present study comprise 30 paranoid SCZ patients (15 males and 15 females), 30 undifferentiated SCZ patients (15 males and 15 females), and 30 healthy controls (15 males and 15 females). The details of their demographics and clinical information were shown in Table 1. SCZ patients were collected from Ningbo Kangning Hospital in Zhejiang province of China. Healthy controls were the volunteers in Ningbo Kangning Hospital. The mean age of SCZ patients was 29.6±5.4 years compared with 30.4±4.0 years for the control subjects. All patients were independently examined by at least two experienced psychiatrists (JC and SG). DSM-IV criteria and SCID-I were used for the diagnosis of SCZ patients. SCZ patients with history of serious or unstable medical illness were excluded from this study. Patients with other psychiatric co-morbidities and drug abuse were also excluded from current study. All SCZ subjects were previously pharmacologically treated by one type of antipsychotic drug. SCZ subjects were prescribed antipsychotic drugs for at least 8 weeks. Healthy controls were age- and gender-matched healthy persons without any history of psychiatric diseases or disorders, and serious diseases such as cancers and cardiac diseases. In addition, we performed a longitudinal research to study the effect of risperidone therapy on the *DRD4* DNA methylation level in peripheral blood. Blood of five additional newly onset male patients were drawn before and after risperidone (3–6 mg/d) treatment as the main antipsychotic medication for 8 weeks. Chemistry Analyzer (AU2700, Olympus, Japan) was used for the measurement of cysteine at the Clinical Chemistry Laboratory of Ningbo Kangning Hospital. The study protocol was approved by the ethical committee of Ningbo University. The informed written consent was obtained from all subjects. The informed written consent of the participants, whose capacity to consent was compromised, were obtained from their guardians and the scientific work complies with the current laws of China.

**Table 1:** The biological and clinical characteristics of 60 SCZ subjects.

Characteristics	Mean ± SD	Range	Men (n = 30)	Women (n = 30)	*p* value
Age(year)	29.6±5.4	[19–41]	29.8±4.0	29.4±6.6	0.686
Occurrence age (year)	21.6±4.8	[12–33]	21.5±4.9	21.7±4.8	0.895
Course of disease (year)	8.0±5.9	[0.2–24.0]	8.5±5.6	7.6±6.4	0.576
Familial history (yes/no)	16/44	/	6/24	10.0/20	/
Mental trauma (yes/no)	20/40	/	7/23	13/17	/
Marriage (yes/no)	20/40	/	6/24	14/16	/
Smoking (yes/no)	11/49	/	11/19	0/30	/
Diagnostic types (paranoid/undifferentiated)	30/30	/	15/15	15/15	/
Outbreak form (chronic/sub-chronic/sub-acute/acute)	16/33/3/8	/	1/24/2/3	15/9/1/5	/
Cysteine (µmol/L)	15.44±12.88	[5.50–64.20]	17.98±15.01	12.89±9.95	0.156
Mean methylation level (%)	69.82±16.76	[28.00–94.20]	71.87±13.33	67.34±15.82	0.210

### Symptomatology assessments

A series of tests were used for symptomatology assessments. These tests comprised the Positive and Negative Syndrome Scale (PANSS), Clinical Global Impressions (CGI), Wisconsin Card Sorting Test, and Chinese version of Wechsler Memory Scale (WMS) test. As an endophenotype for evaluating the cognitive function, p300 wave was also recorded in this study and analyzed by two experienced psychiatrists (JC and SG) by using the neural electricity device (NDI-200P+, Shanghai, China).

### DNA methylation assay

**Figure 1:**
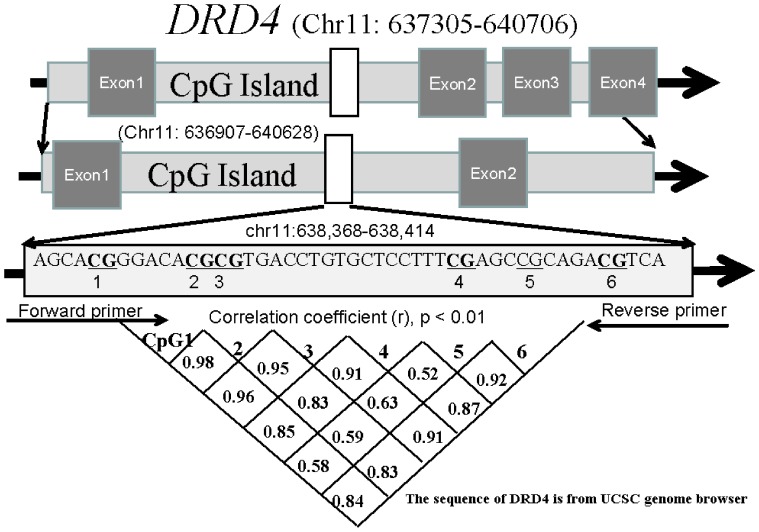
Significant methylation correlation of 6 CpG sites on *DRD4* CpG islands^a^. a: The hg19 version from UCSC genome browser was used for the genomic positions.

Human genomic DNA was prepared from peripheral blood samples using the nucleic acid extraction automatic analyzer (Lab-Aid 820, Xiamen City, China). DNA was quantified using the PicoGreen® double strand (dsDNA) DNA Quantification Kit (Molecular Probes, Inc. Eugene, USA) and extracted from the fresh blood after being drawn from the involved individuals into a vacuum tube with 3.8% Sodium Citrate agent. Then the left blood and extracted DNA were stored at −80°C. As described previously [22], [23], bisulphite pyrosequencing technology was used to determine methylation levels of 6 CpGs on the fragment within *DRD4* CpG island (Figure 1). PCR and pyrosequencing primers for *DRD4* gene amplification were described in Table S1. More details on the pyrosequencing assay are available in the study by Guay et al [24].

### Gene expression assay

Total RNA was extracted from peripheral blood. RNA was extracted with TRIZOL reagent (Invitrogen Life Technologies Co, USA) according to the manufacturer's protocol. RNA isolation and reverse transcription were described in our previous study [25]. Primers for qRT-PCR were designed according to the previous study [26]. The qRT-PCR reactions were conducted according to the previous workflow [25] under the thermal conditions reported before [26]. GAPDH was used as an endogenous control.

### Statistical analysis

Categorical variables were compared using a Pearson chi-square test, whereas Student *t*-test was used to compare the mean group differences for continuous variables. A two-sided P-value <0.05 was considered statistically significant. All statistical analyses were performed with PASW Statistics 18.0 software (SPSS, Inc., Somers, NY, USA).

## Results

A total of 60 SCZ patients and 30 age- and gender-matched healthy controls are recruited in the current association study. The biological information of all SCZ subjects was shown in Table 1. In this study, we have selected a locus containing 6 CpG dinucleotides to explore the association of *DRD4* methylation with SCZ (Figure 1). We found DNA methylation levels were well correlated among all the CpGs (Figure 1, r>0.5, P<0.01), therefore average methylation was used to represent *DRD4* methylation in the following analyses. In addition, we didn't observe any non-CpG methylation in the sequenced fragment.

**Figure 2:**
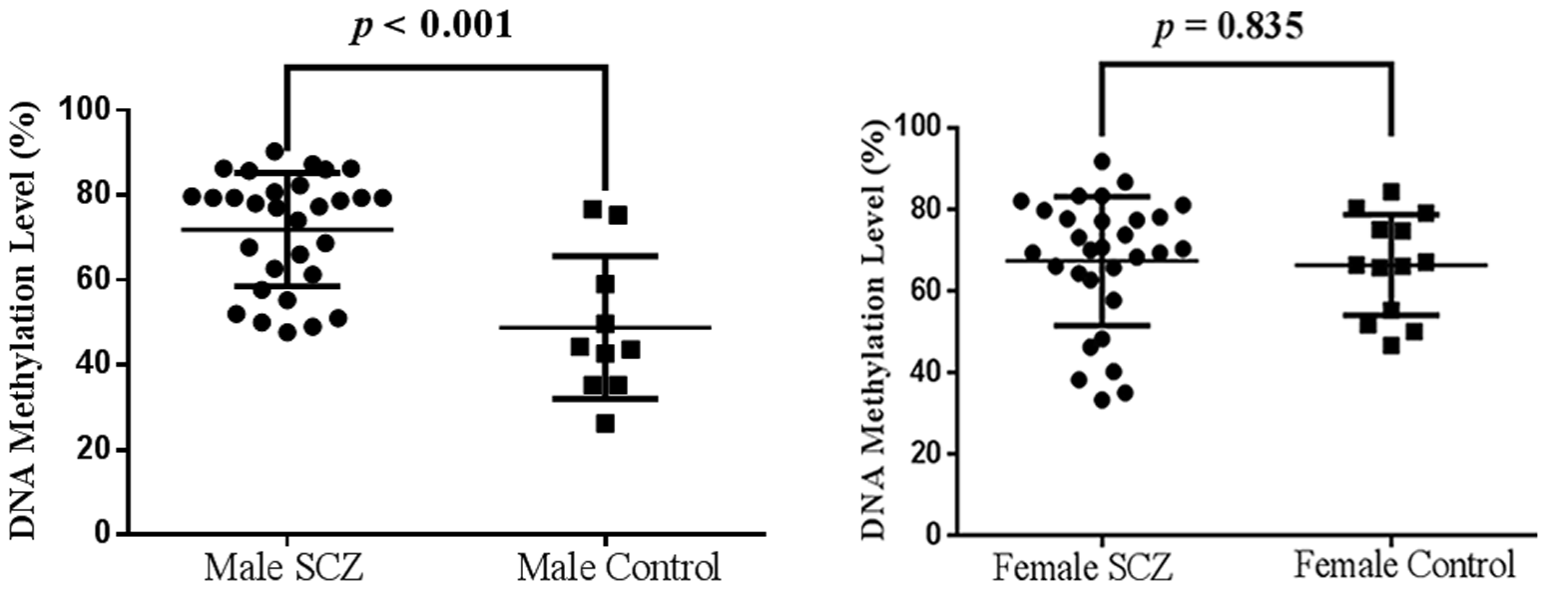
Male specific association of *DRD4* methylation with the susceptibility of SCZ.

**Figure 3:**
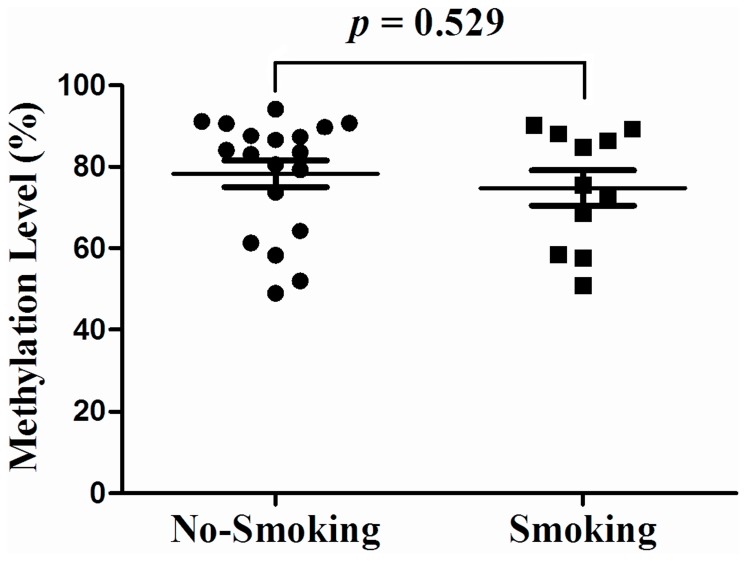
No influence of smoking was found on *DRD4* methylation in male SCZ subjects.

**Figure 4:**
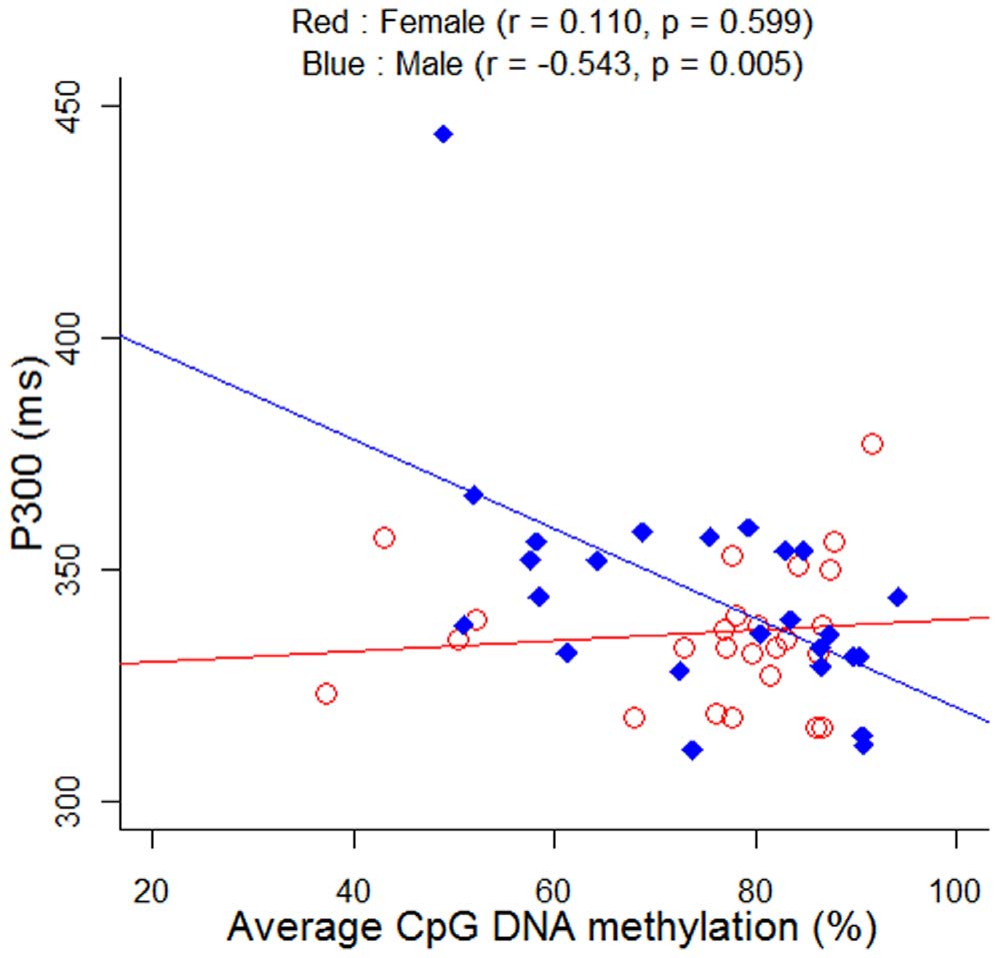
Male specific correlation between *DRD4* methylation and p300 in SCZ patients.

**Figure 5:**
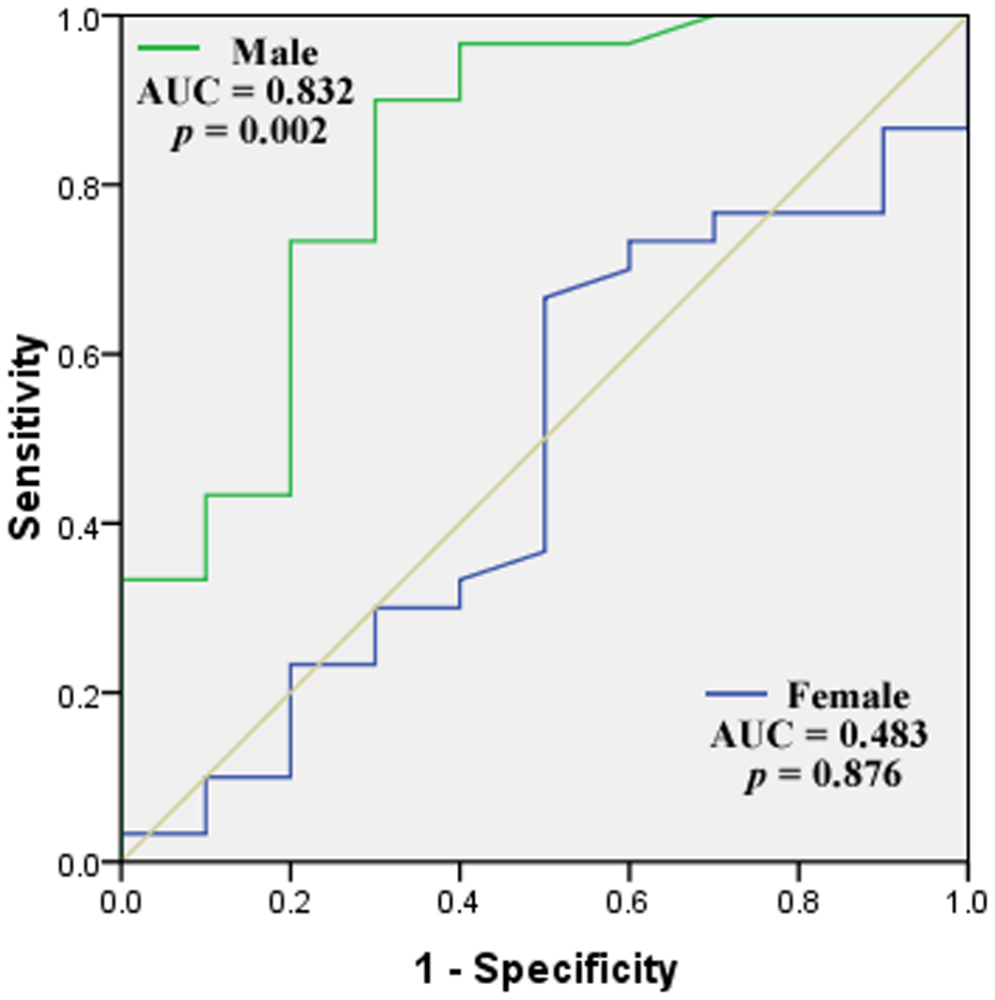
Male specific prediction of SCZ using *DRD4* methylation as a diagnostic marker.

No significant difference of methylation level was found between male (71.87±13.33%) and female (67.34±15.82%) SCZ patients (P = 0.210). Symptomatology assessments of 60 SCZ subjects were shown in Table S2. As shown in Figure S1, no significant difference of *DRD4* methylation was found between paranoid SCZ and undifferentiated SCZ in males (P = 0.986), females (P = 0.591) and combined groups (P = 0.670). Thus, we combined the SCZ subtypes in the following analyses. There was significant difference between SCZ patients (69.82±16.76%) and healthy controls (62.92±19.96%) in *DRD4* methylation (P<0.001). As shown in Figure 2, a breakdown analysis by gender indicated there was significantly higher *DRD4* methylation in male SCZ patients comparing with healthy male controls (male cases versus male controls: 71.87±13.33% versus 48.83±16.79%, P<0.001), however, no difference was observed between female SCZ patients (67.34±15.82%) and female healthy controls (66.31±12.30%, P = 0.835, Figure 2). Hypomethylation of *DRD4* was observed in the male controls (48.83±16.79%) compared with the female controls (66.31±12.30%, P<0.05). There is no difference in *DRD4* methylation levels between smokers and non-smokers in male SCZ patients (P = 0.529, Figure 3). *DRD4* methylation was significantly inversely correlated with p300 in the male SCZ patients (Figure 4, r = −0.543, P = 0.005), but not in the female SCZ patients (Figure 4, r = 0.110, P = 0.599). Meanwhile, receiver operating characteristic (ROC) curve showed that *DRD4* methylation was able to predict the status of SCZ in males [Figure 5, area under curve (AUC) = 0.832, P = 0.002] but not in females (Figure 5, AUC = 0.483, P = 0.876).

**Figure 6:**
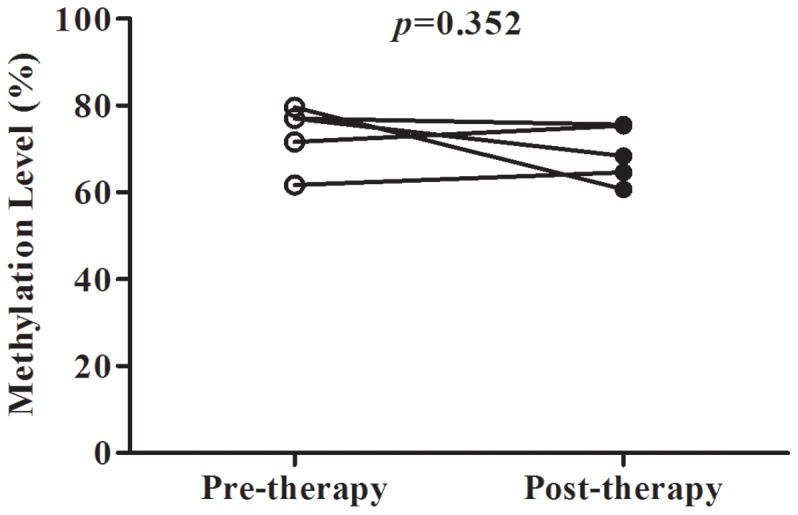
*DRD4* methylation levels in male SCZ patients between before and after risperidone therapy.

An analysis by therapeutic antipsychotics showed that there were no significant differences in *DRD4* methylation among SCZ patients with different drug treatment (Figure S2, P = 0.080). *DRD4* methylation of subgrouped SCZ patients by therapeutic antipsychotics was significantly higher than that of healthy male controls (Figure S3, P<0.05), in contrast that no difference of *DRD4* methylation was observed between female SCZ patients treated by clozapine and healthy female controls (Figure S3, P>0.05). To note, there were significant differences between medicated SCZ males on quetiapine and control males (Figure S3, P<0.001) and significant differences between medicated SCZ females on quetiapine and control females (Figure S3, P = 0.003), although both have equally small numbers of SCZ subjects (4 males and 5 females). Future work is needed to investigate the effect of quetiapine on *DRD4* methylation in SCZ patients. A longitudinal research demonstrated there was no significant different *DRD4* methylation between pre-therapy and post-therapy of risperidone in five additional male SCZ patients (Figure 6, P = 0.352).

In addition, we explored the result of combining the data of this five additional schizophrenic samples with other male controls, the result was accorded with Figure 2 (Figure S4, P<0.001). Furthermore, we have done an association analysis between cysteine levels and *DRD4* methylation in both male and female SCZ subjects. The result showed no correlation between cysteine levels and *DRD4* methylation in both genders of SCZ subjects (Figure S5, Female: r = −0.125, p = 0.509; Male: r = 0.293, p = 0.116). The p300 remained significant after excluding one male sample that was very different from others (Figure S6, Male: r = −0.427, p = 0.037).

**Figure 7:**
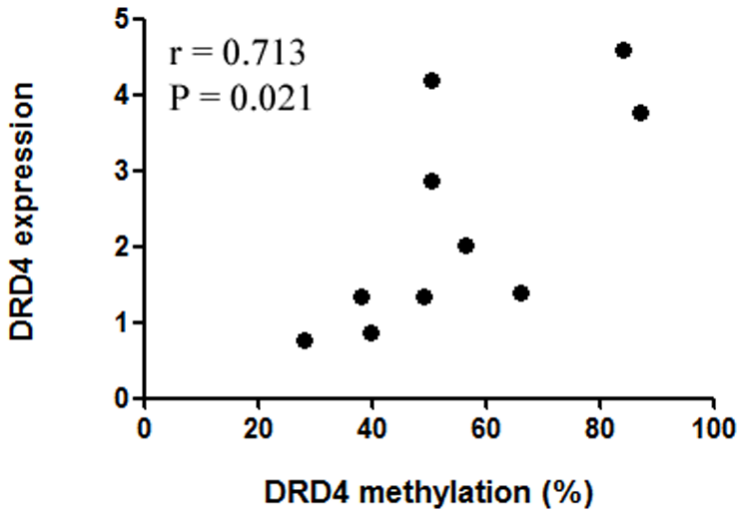
Correlation of *DRD4* methylation levels with *DRD4* gene expression in the healthy males.

In order to investigate the role of *DRD4* methylation in the gene regulation, we further performed expression experiment among 10 male controls. Our results showed a significant positive correlation between *DRD4* methylation and *DRD4* gene expression in the healthy males (r = 0.713, P = 0.021, Figure 7). Our expression data suggests that *DRD4* methylation is functionally relevant to expression level.

## Discussion

SCZ is a type of complex disorder that is contributed by both genetic and environmental factors [27], [28]. As reversible and heritable modifications, epigenetic marks such as DNA methylation are important to reflect the interactions between genetic factors and environmental exposures, and thus may provide us novel understandings for the development of complex diseases or disorders [29], [30].

DA transmitter plays an important role in the psychological function, and the DA receptors are involved in the neural signal pathway modulating human behavior [15], [16]. The dopaminergic system is involved in many kinds of human behavioral performances, such as aggression, delinquency and other behavioural problems [16]. The dysregulation of DA system pathway was hypothesized to be one of major causes of SCZ [31]. Dopaminergic system was the biological targets of psycholytic drugs in SCZ treatment. Genetic variations of genes in this system can influence the response to antipsychotic treatment [10], [17].

*DRD4* gene encodes a subtype of DA receptor that regulates many neurological processes connecting with psychiatric disorders [15]. Genetic association of *DRD4* gene has been reported in SCZ research [29], [32], [33]. However, the role of *DRD4* methylation in SCZ development is not known yet. In the present study, we found that *DRD4* was likely to exert its role in the susceptibility of male SCZ through its aberrant methylation. This finding may provide potential implication for future SCZ diagnosis and the development of personalized antipsychotic therapy.

Gender differences were observed in the onset and prevalence of SCZ [7]. Male patients have a 5-year younger age of onset and have 40% greater risk than the females [7]. Estrogenic protection may contribute to the prevention of SCZ onset in females [34], but the exact reason that causes this gender difference always puzzles SCZ researchers. The gender difference in the SCZ development suggested estrogen had a protective effect against SCZ [35]. Several dopaminergic system genes have been proved to contribute to the etiology of SCZ [36]. *DRD4* may be a member of these susceptibility genes. A *DRD4* variant was shown to be associated with gender-specific abnormal behaviors [16]. Higher exposure to psychosocial risk factors of male individuals may help explain this phenomenon [16]. In addition, we observed that *DRD4* methylation was associated with p300 in males. However, no association was shown between *DRD4* methylation and cytosine level. Our results revealed a male-specific DNA methylation of *DRD4* gene and a positive correlation between *DRD4* methylation and *DRD4* expression in the healthy males. These findings may help to understand the gender disparity in SCZ development. It also indicates that the therapeutic schedule of SCZ needs to be prescribed in a gender-specific way.

There are several limitations in our study that need to be taken with caution. Firstly, DNA methylation levels change across different tissues [37]. Here we use the *DRD4* methylation in peripheral blood as a surrogate of brain tissue. The aberrant blood *DRD4* methylation in male SCZ patients may not imply an aberrant *DRD4* methylation in their brain tissues. Secondly, due to the strict matching criteria for the methylation study, we only recruited 30 paranoid SCZ patients, 30 undifferentiated SCZ patients, and 30 age- and gender-matched healthy controls. Future validation with larger sample size is warranted in other ethnic populations. Thirdly, we didn't investigate how a hypermethylation of *DRD4* gene body activated gene expression. Future work will be needed to take this into account.

In summary, we found a gender dimorphism of *DRD4* methylation in the susceptibility of SCZ. This epigenetic modification may give hints to elaborate the pathological mechanisms of SCZ, and provide new biological aspects of *DRD4* gene. The aberrant methylation of *DRD4* gene may be a valuable gender-specific biomarker to monitor the risk and development of SCZ.

## Supporting Information

Table S1 **Primers for** ***DRD4*** **methylation analysis.**
(DOC)

Table S2 **Symptomatology assessments of 60 SCZ subjects in a series of tests.**
(DOCX)

Figure S1 **Minimal difference of** ***DRD4*** **methylation between the two subgrouped SCZ patients.**
(TIF)

Figure S2 **Stratification test by the antipsychotic medication in SCZ.**
(TIF)

Figure S3 Breakdown analyses of stratification test by therapeutic antipsychotics.* *: Only SCZ patients were on medication.
(TIF)

Figure S4 **Analysis of combining the data of these five additional schizophrenic samples with other male controls.**
(TIF)

Figure S5 **Association analysis between cysteine levels and** ***DRD4*** **methylation in both male and female SCZ subjects.**
(TIF)

Figure S6 **Male specific correlation between** ***DRD4*** **methylation and p300 in SCZ patients after removing one male with large value.**
(TIF)
