# Supplementary material for: Male-Specific Association between Dopamine Receptor D4 Gene Methylation and Schizophrenia
Source: PLoS One. 2014 Feb 19;9(2):e89128. doi: 10.1371/journal.pone.0089128 (PMC3929639; doi:10.1371/journal.pone.0089128)
Supplement: Table S1 — Primers for DRD4 methylation analysis. (DOC) [file pone.0089128.s001.doc]

Supplemental Table 1: Primers for *DRD4* methylation analysis

| Forward primer: | 5’- GTGAATTTAGGAGGTTGGGGTAGA -3’ |
| --- | --- |
| Reverse primer: | 5’-Biotin- CAAAAAAACAAACAACCCCTCTAA -3’ |
| Sequencing primer: | 5’- TTGGGGTAGAGATTAGT -3’ |
