# Supplementary material for: Male-Specific Association between Dopamine Receptor D4 Gene Methylation and Schizophrenia
Source: PLoS One. 2014 Feb 19;9(2):e89128. doi: 10.1371/journal.pone.0089128 (PMC3929639; doi:10.1371/journal.pone.0089128)
Supplement: Table S2 — Symptomatology assessments of 60 SCZ subjects in a series of tests. (DOCX) [file pone.0089128.s002.docx]

Supplemental Table 2: Symptomatology assessments of 60 SCZ subjects in a series of tests

|  | **Mean ± SD.** | **Men**  **(n=30)** | **Women**  **(n=30)** |
| --- | --- | --- | --- |
| **PANSS** |  |  |  |
| Total score | 55.87±15.56 | 58.37±.12.31 | 53.37±.12.26 |
| Positive scores | 11.67±6.18 | 10.67±5.07 | 12.67±5.10 |
| Negative scores | 12.07±5.52 | 13.57±4.13 | 10.57±5.32 |
| General pathologic score | 29.98±9.69 | 32.48±6.35 | 27.48±5.45 |
|  |  |  |  |
| **CGI** |  |  |  |
| SI | 2.94±1.03 | 3.19±1.26 | 2.69±1.12 |
| GI | 2.20±1.28 | 1.95±0.73 | 2.45±0.71 |
| EI | 1.78±1.03 | 1.91±0.78 | 1.65±0.57 |
|  |  |  |  |
| **WCST** |  |  |  |
| Categories achieved | 4.83±1.32 | 4.73±1.13 | 4.93±1.22 |
| Correct | 50.53±10.54 | 50.02±10.51 | 51.03±9.01 |
| Error | 63.96±12.72 | 61.96±20.70 | 65.96±24.10 |
| Perseverative error | 46.30±21.04 | 43.70±21.13 | 48.90±24.19 |
| Nonperseverative error | 16.73±6.20 | 16.21±6.27 | 17.24±7.10 |
|  |  |  |  |
| **WMS** |  |  |  |
| MQ | 76.89 ±17.68 | 77.89 ±19.18 | 75.89 ±20.01 |
| Experience | 4.24 ±1.02 | 4.09 ±1.01 | 4.39 ±1.03 |
| Orientation | 4.50 ±1.21 | 4.62 ±1.16 | 4.37 ±1.05 |
| 1-100 | 8.02 ±2.26 | 8.51 ±4.03 | 7.53 ±3.23 |
| 100-1 | 8.73 ±2.23 | 9.26 ±3.97 | 8.20 ±2.37 |
| Accumulation | 8.25 ±3.86 | 7.75 ±2.29 | 8.75 ±3.20 |
| Card | 5.82±3.27 | 5.57±2.01 | 6.07±2.51 |
| Recognition | 5.73±3.98 | 6.13±2.79 | 5.33±2.19 |
| Reproduction | 6.98±4.37 | 7.68±4.06 | 6.28±3.06 |
| Association | 6.85±4.64 | 7.15±3.43 | 6.55±3.63 |
| Touch | 6.17±1.87 | 6.02±1.72 | 6.32±1.32 |
| Comprehension | 6.44±3.47 | 6.29±3.15 | 6.59±3.55 |
| Number Memory | 7.38±3.67 | 7.83±4.07 | 6.93±3.02 |
