# Supplementary figures and images for: Male-Specific Association between Dopamine Receptor D4 Gene Methylation and Schizophrenia
Source: PLoS One. 2014 Feb 19;9(2):e89128. doi: 10.1371/journal.pone.0089128 (PMC3929639; doi:10.1371/journal.pone.0089128)

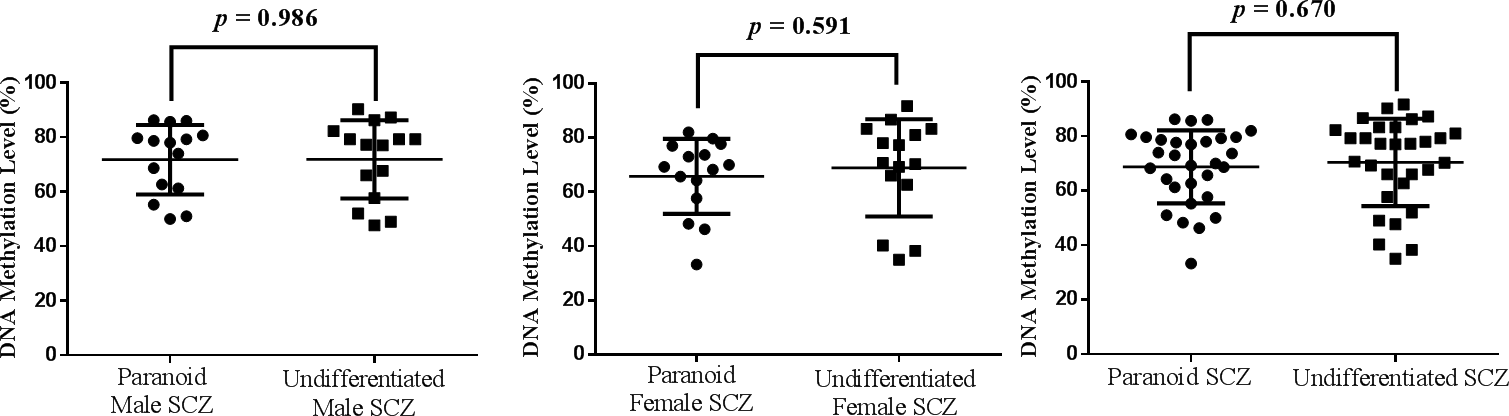

Supplement: Figure S1 — Minimal difference of DRD4 methylation between the two subgrouped SCZ patients. (TIF) [file pone.0089128.s003.tif]

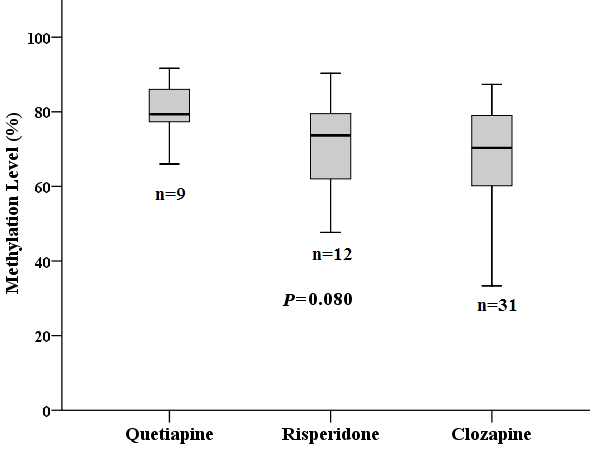

Supplement: Figure S2 — Stratification test by the antipsychotic medication in SCZ. (TIF) [file pone.0089128.s004.tif]

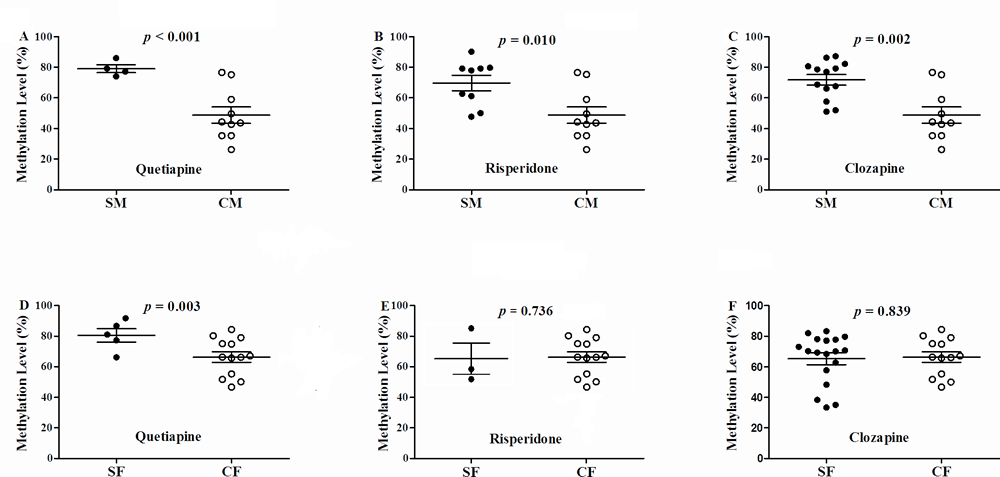

Supplement: Figure S3 — Breakdown analyses of stratification test by therapeutic antipsychotics.* *: Only SCZ patients were on medication. (TIF) [file pone.0089128.s005.tif]

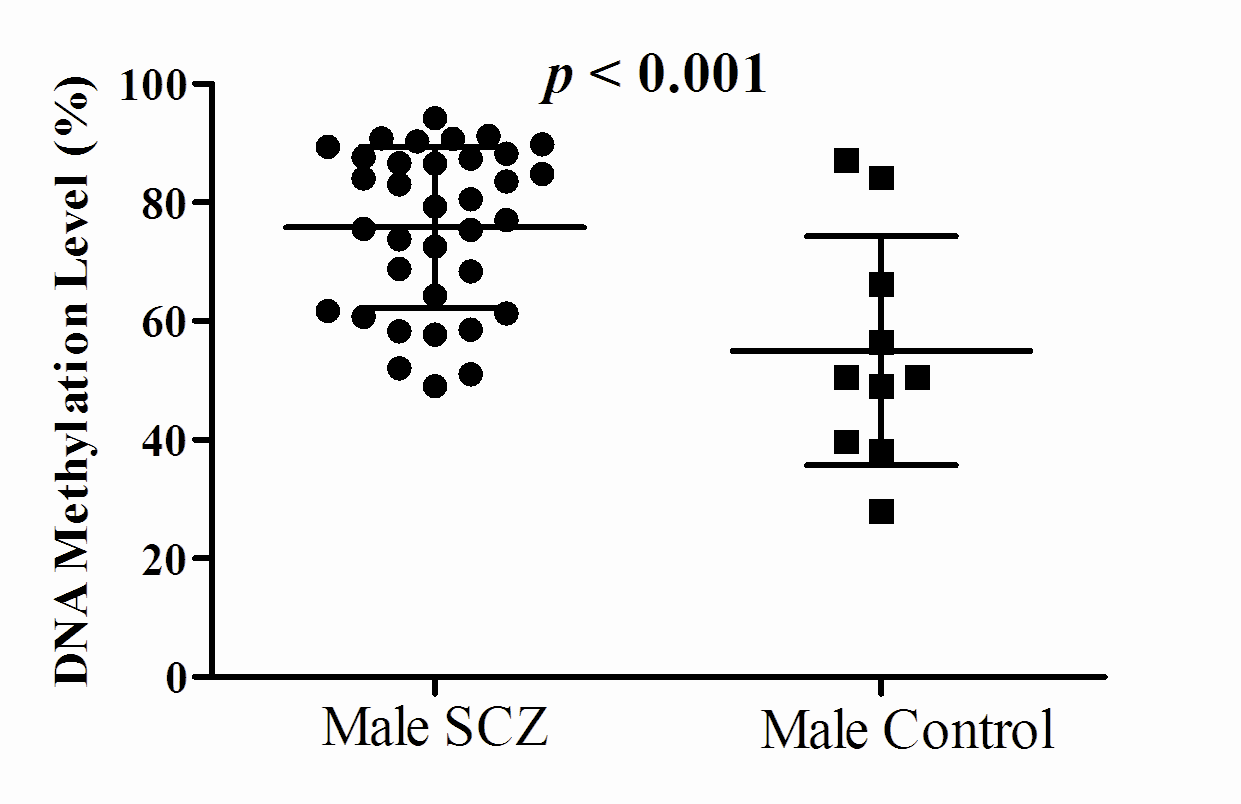

Supplement: Figure S4 — Analysis of combining the data of these five additional schizophrenic samples with other male controls. (TIF) [file pone.0089128.s006.tif]

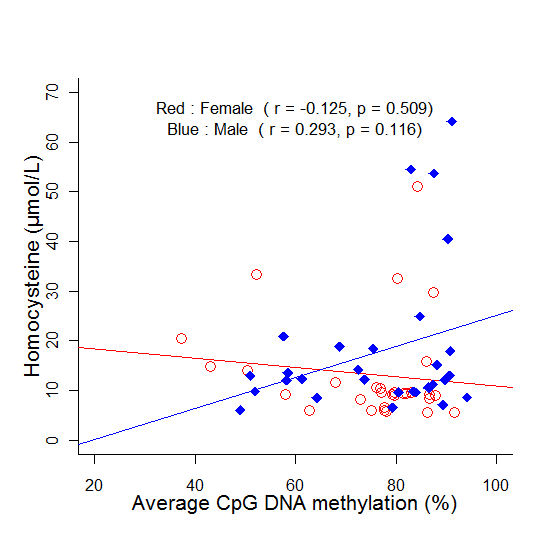

Supplement: Figure S5 — Association analysis between cysteine levels and DRD4 methylation in both male and female SCZ subjects. (TIF) [file pone.0089128.s007.tif]

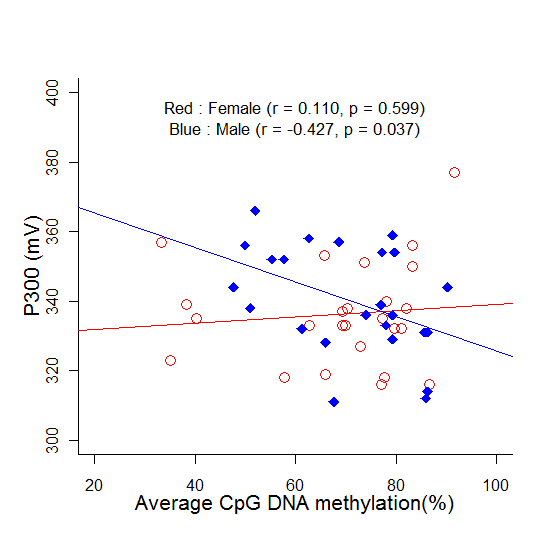

Supplement: Figure S6 — Male specific correlation between DRD4 methylation and p300 in SCZ patients after removing one male with large value. (TIF) [file pone.0089128.s008.tif]
